# Social inequalities in women exposed to obstetric and gyneco-obstetric violence in Ecuador: a cross-sectional study

**DOI:** 10.1186/s12905-022-01998-2

**Published:** 2022-10-13

**Authors:** Fara Faith Arias Fuentes, Erika Arteaga, Miguel San Sebastián

**Affiliations:** 1 Dept. of Epidemiology and Global Health, Umeå University, 901 87 Umeå, Sweden; 2 Health Sciences College, University of San Francisco de Quito, Quito, Ecuador

**Keywords:** Obstetric, Gynecological, Violence, Social inequalities, Ecuador

## Abstract

**Background:**

Obstetric and gyneco-obstetric violence (OV, GOV) is a concerning public health problem, particularly in Latin America. This study aimed to determine the prevalence of OV and GOV and to assess its socio-geographical distribution in Ecuador.

**Methods:**

This cross-sectional study used data from a national survey conducted in 2019 (n = 17,211) among women aged 15 years and over. Independent variables included age, marital status, education, ethnicity, place of residence and region. The chosen outcomes were lifetime experience of OV and GOV. Frequency tables were calculated and crude and adjusted regression models estimating prevalence ratios and their 95% confidence intervals were computed.

**Results:**

Nearly one-third (32.8%) of the participants had experienced OV and two-fifths (41.86%) GOV at least once in their lifetime. Prevalence of OV were particularly common in women 26–35 and 46–55 years old, with primary or middle education and in urban regions. In comparison, GOV had a higher prevalence in women aged > 65 years and with no formal education. Both subtypes of violence were more common among women with current or earlier partners compared with the single ones. Also the two outcomes were more prevalent in the non-white population, OV among the populations of colour (POC), while GOV both, in the POC and Indigenous group. Additionally, women from the Highlands and Amazon reported higher OV and GOV than the Coastal group.

**Conclusion:**

Our study showed that OV and GOV are common in Ecuador and identified an unequal distribution of their prevalence across different socio-geographical groups. Further studies including more social factors and a continuous monitoring of OV and GOV are recommended. Current policies, laws to protect women and guidelines regarding the treatment of women, particularly in health care settings, need to be constantly advocated for and effectively implemented in the country.

## Background

Obstetric violence (OV) is a relatively new term in public health, first stated in Venezuela in the 2007 “Organic Law on Women’s Rights to a Violence-Free Life” [1]. OV has been described as “the appropriation of the body and reproductive processes of women by health personnel, which is expressed as dehumanized treatment, an abuse of medication, and to convert the natural processes into pathological ones, bringing with it loss of autonomy and the ability to decide freely about their bodies and sexuality, negatively impacting the quality of life of women” [1]. Essentially, OV involves any disrespectful, discriminatory, negligent, reckless or omissive treatment against women during childbirth, which, as the World Health Organization (WHO) states, violates women’s rights to respectful care and threatens their rights to life, health and physical integrity [2, 3].

This type of conduct has been revealed and acknowledged in different studies worldwide, referring to examples of OV such as physical and psychological violence, verbal abuse and profound humiliations occurring during women´s health care visits. Other examples given include procedures performed without consent or with limited information or coerced, such as C-sections, episiotomies, sterilization or vaginal examinations [2, 4–8]. Denial of care, lack of privacy, refusal of treatment and admission to health facilities and threats of child apprehension have also been described as examples of this type of violence [2, 4, 9, 10].

OV may occur during obstetric procedures as well as during pre-and post-partum care (the so-called gyneco-obstetric violence (GOV), leading not only to psychological ill-health, but also contributing to the development of complications, significantly affecting maternal morbidity and mortality and fetal and child development [3, 11].

The abuse of women within the healthcare system has been often spoken about but largely undocumented until recently. In a cross-sectional study conducted in four African and Asian countries (Ghana, Guinea, Nigeria and Myanmar), around 42% of women reported that they had experienced physical or verbal abuse or discrimination during childbirth at a health center [7]. Other studies covering abuse during delivery have found a prevalence of 74% in Ethiopia, 19–28% in Tanzania, 28.8% in India, 17% in the U.S., 21.2% in Italy and 38% in Spain [5, 8–10, 12, 13]. A study covering the prevalence of abuse in health care associated with OV in six European countries (Belgium, Iceland, Denmark, Estonia, Norway and Sweden) showed a prevalence of one in five pregnant women attending routine antenatal care [14]. Studies from Latin America have also observed an OV prevalence of 29–33% in Mexico and 12.6% in Brazil during childbirth [6, 15, 16].

This high prevalence of OV among different studies in diverse contexts reveals this type of violence as a common problem worldwide. While these figures are difficult to compare and interpret between countries and regions due to different methodologies and settings, common social factors associated with a higher or lower likelihood of mistreatment at healthcare have been identified. For instance, previous studies have found correlations between social factors related to stigmatization, such as a young age, low social-economic status, lower education level, unmarried status, belonging to an ethnic or religious minority or being HIV positive and OV [2, 5, 7, 8, 13, 15, 17, 18].

Ecuador has continuously made efforts to protect women from violence during the last three decades. The country implemented the “Law of Rights and Protection of the Patient” in 1995 and the “Organic Law on the Integrated Criminal Code” in 2014, covering different definitions of violence and specific penalties for violence against women [19, 20]. Additionally, recently efforts have been made to create a legal framework to prevent, eliminate and punish all forms of violence against women. For instance, the “Comprehensive Organic Law to Prevent and Eradicate Violence against Women” was created in 2018, including both OV and GOV as its scope of violence. As part of this framework and responding to the obligation of collecting information on gender-based violence, the Ecuadorian National Institute of Statistics and Census (INEC in Spanish) performed the National Survey of Family Relations and Gender Violence against Women in Ecuador (ENVIGMU in Spanish) in 2019 [21]. For the first time, the prevalence for OV and GOV were measured. By doing so, evidence was provided as a tool for reinforcing and improving policies regarding the treatment of women in health care settings [21].

While descriptive results of this survey have been published [21], a systematic analysis of the distribution of OV across different socio-geographical factors has not been made. Therefore, this study aimed to determine the prevalence of OV and GOV and assess the socio-geographical inequalities in the exposure to these types of violence in Ecuador.

## Methods

### Study design

This cross-sectional used secondary data publicly available from the second National Survey of Family Relations and Gender Violence against Women conducted in Ecuador in 2019 [21]. This survey aimed to assess the prevalence of different types of violence against women, including OV and GOV, in a representative sample of 17,211 women aged 15 years and older.

A three-stage cluster sampling obtained national, regional, rural, and urban population estimates. Previous estimates of violence were used to calculate the sample size, using an error rate of 5% and a non-response rate of 5% [22]. It resulted in a selection of 24,427 households, including 27,842 women, from which one woman per household was selected randomly, resulting in a total of 19,161 women. The final study was composed of 17,211 women (89.92%) due to lack of response, such as not wanting to participate or absence from home. The collected data mainly contains information on different dimensions and types of violence against women and specific sociodemographic characteristics.

### Measures

#### Dependent variables

All questions for this study refer to the reported experiences of OV and GOV at least once during the participants’ lifetime. These questions referred to gynaecological and pre-partum visits as well as during childbirth and post-partum visits. Women who answered “yes” to at least one question referring to each type of violence were considered to have been exposed to violence. The variables´ coding is provided by the Ecuadorian National Institute of Statistics and Census in its webpage [21].

#### Obstetric violence

OV was characterized by verbal and physical violence, and non-consensual care/procedures violating the woman’s autonomy during child labour or post-partum. Exposure to experiences associated with verbal violence included acts such as: yelling, scolding, insulting, criticizing, humiliating, or threatening not to help upon complaints, being ignored or denied information upon childbirth or post-partum. Other examples associated with physical violence were being subjected to the demand of shaving of pubic hair or performing an enema, vaginal examinations performed repeated times and by different individuals without consent or information, performed Kristalleur manoeuvre or given any medication to accelerate the labour, performed episiotomy during labour without explanation or suture after episiotomy without local anaesthesia. Lastly, non-consensual care/procedures covered in the questions included the denied choice of accompaniment during and after childbirth, the denied choice of birth position, denied alternative to pain medication without any explanation, keeping the mother from seeing, carrying, or breastfeeding the baby immediately after birth without explaining the reason to the delay, asked the third person for authorization regarding sterilization for the patient, and lastly any administered contraceptives/operation/sterilization without consent or by being pressured into it.

#### Gyneco-obstetric violence

GOV was similarly characterized, covering experiences associated with verbal and physical violence as well as non-consensual care during gynecological and pre-partum visits. Questions describing verbal violence included sexual comments/insinuations that made the patient offended, humiliated, or uncomfortable during the visit, gestures/comments regarding the individual’s sexual activities, and reproductive choices. Experiences regarding non-consensual care and physical violence were also covered. Examples included the denied choice of accompaniment during a medical visit, performing vaginal examinations without informed consent and in the presence of a third person without any explanation, realized pap smear, pelvic examination, mammography, or other examination without informed consent, administered contraceptives without information regarding side effects. Questions related to abortion-related experiences were also incorporated: waiting many hours to get help without medical justification, not being given pain medication, feeling interrogated, shamed or threatened, isolated or purposely placed with other women who had children to make the patient feel bad, denied information regarding treatment options, being reported to the police or any justice system.

#### Independent variables

The independent variables were chosen based on data availability and organized, using the social-ecological model of violence, into individual, relationship and societal factors. Two variables from the survey were considered as individual factors: age which was grouped into six categories (15–25, 26–35, 36–45, 46–55, 56–65 or > 65 years of age), and education, grouped into four: no education, primary/middle, secondary and university or higher. The survey included a question on financial aid, but only 1437 women answered the question and, therefore, was excluded from the analysis. Marital status was the only relationship-level factor available and recoded into married, separated/divorced/widowed, living with partner and single. Ethnicity and place of residence were included as societal factors. Ethnicity was recoded into Indigenous, people of color (POC) (afro-Ecuadorian and mulato), mestizo (including montubio), white and others. Since ethnicity includes a solid cultural dimension, it was categorized as a societal factor. Place of residence was captured with two variables: area, which was grouped into rural and urban; and region, divided into four categories: Coast, Highlands, Amazon or the Galapagos and others (undefined areas).

### Statistical analysis

Frequency tables and percentages were used to present the descriptive characteristics of the population and the two violence outcomes. Bivariate analyses between the independent variables and the violence outcomes were carried out first. All statistically significant covariates were then included in a multivariable regression model. Prevalence ratios (PR) and their 95% confidence intervals (95% CI) were calculated as the measure of association. In all analyses, sample weighting was applied to adjust for the unequal probability of sample selection and interview. A detailed explanation of the weighting procedure can be found in the Survey Manual [23]. The Huber/Whites/sandwich estimator was also applied to obtain robust standard errors. In addition, collinearity was checked, with all the independent variables having a variance inflation factor (VIF) lower than 1.5. The Stata 14 software was used to conduct the analyses.

### Ethical consideration

All women that participated in the study gave their consent. Since the data is publicly available, no ethical approval was necessary to conduct this study.

## Results

### Description of the study sample

Table 1 presents the characteristics of study participants in total and stratified by violence outcomes. Overall, 17,211 women participated in the study, the majority being between 15 and 25 years old (24.5%), followed by the 36 to 45 age group (17.7%). Most had finished secondary education (40.7%) and one-third (33.1%) belonged to the primary/middle educational group. A similar distribution of the participants across the different civil statuses was found, slightly higher among married women (32.1%). The most common ethnic group in the sample was the mestizo (85.8%) and most of the participants lived in urban areas (71.3%). Regarding the four different regions, the majority lived in the Coast and the Highlands (48.7% and 46.4%, respectively).

**Table 1:** Characteristics of study participants in total and stratified by violence outcomes, Ecuador 2019

	Total sample (%)	Obstetric violence (%)	Gyneco-obstetric violence (%)
**Total sample**	17,211 (100)	5,643 (32.79)	7,173 (41.68)
**Individual**			
**Age (years)**			
15–25	4,213 (24.48)	486 (11.53)	685 (16.26)
26–35	2,816 (16.36)	1,069 (37.97)	1,275 (45.27)
36–45	3,048 (17.71)	1,227 (40.25)	1,439 (47.20)
46–55	2,813 (16.34)	1,201 (42.70)	1,426 (50.71)
56–65	2,270 (13.19)	895 (39.43)	1,151 (50.71)
> 65	2,051 (11.92)	766 (37.31)	1,198 (58.38)
**Education**			
University or higher	3,631 (21.10)	1,087 (29.94)	1,286 (35.41)
Secondary	6,997 (40.65)	2,107 (30.11)	2,423 (34.64)
Primary/Middle	5,699 (33.11)	2,174 (38.15)	2,850 (50.01)
None	885 (5.14)	275 (31.11)	615 (69.54)
**Relationship**			
**Marital status**			
Single	4,555 (26.46)	400 (8.78)	691 (15.16)
Living with partner	3,562 (20.70)	1,354 (38.00)	1,571 (44.11)
Married	5,524 (32.10)	2,459 (40.07)	3,012 (53.21)
Separated/divorced/widowed	3,571 (20.75)	1,431 (44.52)	1,900 (54.53)
**Societal**			
**Ethnicity**			
Indigenous	1,237 (7.19)	388 (31.39)	740 (59.82)
People of color	576 (3.35)	216 (37.46)	258 (44.83)
Mestizo	14,762 (85.77)	4,871 (33.00)	5,963 (40.39)
White and others	635 (3.69)	168 (26.49)	212 (33.44)
**Area**			
Rural	4,935 (28.68)	1,520 (30.80)	2,301 (46.61)
Urban	12,275 (71.32)	4,124 (33.59)	4,873 (39.70)
**Region**			
Coast	8,382 (48.70)	2,362 (28.17)	2,787 (33.25)
Highlands	7,984 (46.39)	3,026 (37.90)	3,997 (50.07)
Amazon	789 (4.58)	241 (30.49)	372 (47.21)
Galapagos and others	56 (0.32)	16 (27.77)	17 (30.83)

A total of 5,643 (32.79%) women reported having experienced OV, alongside 7,173 (41.68%) women that reported GOV. Regarding age, the highest prevalence of OV was seen in the age group of 46–55 years old (42.70%) and 36–45 years old (40.25%), while GOV had a higher prevalence in the age group of > 65 years old (58.38%). Women in the primary/middle education group reported a higher prevalence of exposure to OV (38.15) and those in the none education group for GOV (69.54%) compared with the other education groups. Regarding civil status, separated/divorced/widowed women reported the highest prevalence of both types of violence, 44.52% for OV and 54.53% for GOV. Concerning ethnicity, the POC population constituted the highest group reporting exposure to OV (37.46%) and the Indigenous population for GOV (59.82%). Finally, a higher prevalence of OV was also observed in the participants living in urban areas (33.59%) and the Highlands region (37.90%). A slightly different pattern was seen for GOV, with a prevalence of 46.61% in rural areas and 50.07% in the Highlands region.

### Regression analyses

The results, of regression analyses both crude and adjusted, are presented in Table 2. All independent variables associated with OV were statistically significant except specific subgroups such as secondary education, none education, and the Galapagos and other regions in the crude analysis. Similarly, secondary education and the Galápagos region, were not statistically significantly associated with GOV in the crude analysis.

**Table 2:** Weighted crude and adjusted prevalence ratios with their 95% confidence intervals (95% CI) between the independent variables and the violence outcomes, Ecuador 2019

	Obstetric violence		Gyneco-obstetric violence	
	**Crude**	**Adjusted**	**Crude**	**Adjusted**
**Individual**				
**Age (years)**				
15–25	1	1	1	1
26–35	3.30 (2.99, 3.63)	1.73 (1.45, 2.06)	2.78 (2.57, 3.01)	1.70 (1.46, 1.97)
36–45	3.49 (3.18, 3.84)	1.65 (1.37, 1.98)	2.90 (2.69, 3.14)	1.61 (1.38, 1.88)
46–55	3.70 (3.37, 4.07)	1.73 (1.44, 2.09)	3.12 (2.89, 3.37)	1.74 (1.49, 2.03)
56–65	3.42 (3.10, 3.77)	1.66 (1.37, 2.01)	3.12 (2.88, 3.38)	1.72 (1.47, 2.01)
> 65	3.24 (2.93, 3.58)	1.56 (1.29, 1.92)	3.59 (3.32, 3.88)	1.84 (1.57, 2.15)
**Education**				
University or higher	1	1	1	1
Secondary	1.01 (0.95, 1.07)	1.10 (1.01, 1.20)	0.98 (0.93, 1.03)	1.07 (0.99, 1.15)
Primary/Middle	1.27 (1.20, 1.35)	1.12 (1.02, 1.22)	1.41 (1.34, 1.49)	1.19 (1.11, 1.28)
None	1.04 (0.93, 1.16)	0.83 (0.70, 0.98)	1.96 (1.85, 2.09)	1.31 (1.19, 1.43)
**Relationship**				
**Marital Status**				
Single	1	1	1	1
Living with partner	4.33 (3.91, 4.79)	3.65 (2.97, 4.48)	2.91 (2.69, 3.15)	2.39 (2.04, 2.79)
Married	5.07 (4.60, 5.59)	3.71 (3.01, 4.57)	3.60 (3.35, 3.87)	2.40 (2.05, 2.81)
Separated/divorced/widowed	4.56 (4.12, 5.05)	3.56 (2.87, 4.42)	3.51 (3.26, 3.78)	2.46 (2.10, 2.89)
**Societal**				
**Ethnicity**				
White and others	1	1	1	1
Mestizo	1.25 (1.09, 1.42)	1.21 (0.99, 1.48)	1.21 (1.08, 1.35)	1.15 (0.98, 1.36)
Indigenous	1.19 (1.02, 1.38)	1.10 (0.87, 1.40)	1.79 (1.59, 2.01)	1.33 (1.12, 1.58)
People of color	1.41 (1.20, 1.67)	1.45 (1.15, 1.84)	1.34 (1.16, 1.55)	1.34 (1.10, 1.62)
**Area**				
Rural	1	1	1	1
Urban	1.09 (1.04, 1.15)	1.14 (1.06, 1.23)	0.85 (0.82, 0.88)	1.00 (0.95, 1.05)
**Regions**				
Coast	1	1	1	1
Highlands	1.35 (1.29, 1.41)	1.47 (1.37, 1.58)	1.51 (1.45, 1.56)	1.52 (1.43, 1.61)
Amazon	1.08 (0.97, 1.21)	1.17 (1.07, 1.27)	1.42 (1.31, 1.54)	1.37 (1.28, 1.46)
Galapagos and others	0.99 (0.65, 1.51)	0.99 (0.78, 1.25)	0.93 (0.63, 1.37)	0.90 (0.72, 1.12)

In the adjusted analysis, the prevalence ratio (PR) for OV was evenly distributed amongst the age groups, with the most senior (> 65) age group reporting the lowest (PR: 1.56; 95% CI: 1.29, 1.92) and the age group of 26–35 and 46–55 years old, the highest (PR: 1.73; 95% CI: 1.45, 2.06 and PR: 1.73; 95% CI: 1.44, 2.09 respectively) exposure to violence compared to the youngest reference group. A similar pattern was observed for GOV. However, the oldest group reported the highest exposure to violence in this case (PR: 1.84; 95% CI: 1.57, 2.15).

Slight increases in prevalence were observed in the primary and middle education group in both outcomes, with a decrease in those with no education (PR: 0.83; 95% CI: 0.70, 0.98) in OV but an increase in GOV (PR: 1.31; 95% CI: 1.19, 1.43). As expected, higher prevalence ratios of both types of violence were reported among women who had or had had a partner compared to the single ones, with similar distributions of the effect sizes among different civil statuses but higher in the OV compared to GOV. Regarding ethnicity, OV was more concentrated among the POC (PR: 1.45; 95% CI: 1.15, 1.84) while GOV among POC (PR: 1.34; 95% CI: 1.10, 1.62) and Indigenous populations (PR: 1.33; 95% CI: 1.12, 1.58).

While a statistically significant higher prevalence for OV was also observed in urban in comparison to rural areas (PR: 1.14; 95% CI: 1.06, 1.23), no differences were noticed for GOV. Women from both the Highlands and Amazon regions reported being exposed to OV and GOV more often than the Coastal region, the reference group. The highest prevalence ratio for OV was reported in the Highlands region (PR: 1.47; 95% CI: 1.37, 1.58), followed by the Amazon (PR: 1.17; 95% CI: 1.07, 1.27). A similar pattern was observed regarding GOV, with the Highlands (PR: 1.52; 95% CI: 1.43, 1.61) and the Amazon (PR: 1.37; 95% CI: 1.28, 1.46) regions reporting a higher prevalence ratio.

## Discussion

This study investigated the prevalence and associated factors for OV and GOV in Ecuador using data from a national survey among 17,211 women aged 15 years old and over conducted in 2019. This analysis showed a lifetime prevalence of 32.8% in OV and 41.7% in GOV, respectively.

Considering the different methodologies, time exposure and definitions of OV, to compare with studies around the world is a challenging task. The prevalence of OV and GOV in Ecuador was higher than that reported in studies from Brazil, Tanzania, and high-income countries, ranging from 12.6% in Brazil up to 19–28% in Tanzania [5, 10, 12, 14, 15]. However, a prevalence of 4% has been described in a small sample (n = 379) of Ethiopian women [8]. In several other countries, the prevalence of OV was comparable to Ecuador, ranging from 28.8% India to 42% in other Asian and African countries. Additionally, studies from Spain (38%) [13] and Mexico (33%) [16] have reported exposure to OV similar to this study.

In order to understand why OV is so common and more often present into certain populations, a closer examination of this study’s results has been made, taking into account current, widespread norms of old-fashioned gender roles, structural racism and classism, as well as hierarchal statuses between health care personnel and patients.

While both OV and GOV had a similar distribution among all ages, OV was more common in the 26–35 and 46–55 years-old group, while GOV was more frequent in the > 65. This can be explained by OV being conducted in childbirth and post-partum settings where women between 25 and 55 years old are of childbearing age. For GOV, it was expected a higher likelihood of having attended more gynecological visits in the > 65-years-old group than among the reference group of women aged 15–25 years old.

Women who have not been able to get an education had a lower risk of OV but higher of GOV. The literature supports usually the finding of low education associated with violence [7, 24]. The relationship between lack of education and GOV can be explained by different circumstances. Different healthcare settings often project a patriarchal, non-patient-centered approach. Information is sometimes not given, and if shared, in a limited amount and not adapted to the individual’s education level and knowledge about the topic. Due to circumstances such as urgency and little capacity, consent is often not obtained and thus compromised. This, together with a potential lack of awareness of their rights and social norms, which accept mistreatment from higher instances/hierarchy, may create an obstacle for women to seek help and challenge the abuse [13]. The lower risk of OV among the low educated women in this study would however require further investigation.

Remarkably, some studies have reported expressed annoyance by the healthcare professionals at women’s inability to understand instructions and processes related to pregnancy and childbirth, attributing this as ignorance on the side of low educated and/or poor women [6]. This situation becomes an obstacle for the relationship between the patient and health care professionals. Reassessment of the medical and nursing curriculum and the capacity building in current health care personnel is needed to overcome this hindrance. Awareness for the health care personnel should be implemented, adding to the already existing notion of saving lives without patient mistreatment [6]. Besides self-awareness, training should focus on the ethical aspects of health care, intercultural approaches, and sensitivity towards sexual and reproductive health and rights [25].

Marital status seems to play a role in the risk of exposure to OV and GOV. In this study, both married women and women who had or had had partners were more often subjected to violence. Different scenarios could explain this prevalence, mainly the increased number of visits for obstetric and gynecological services that a woman attends to when having a partner. Besides, violations of the women’s autonomy often include gathering consent from their partner or other relatives, rather than the women herself. In current literature, unmarried women are overrepresented in exposure to OV [2, 15]. In order to understand the role of marital status in relation to OV and GOV, further research is needed.

Overall, non-white participants reported OV and GOV more often, confirming certain structural racism among healthcare professionals. Literature in Latin America regarding associations between ethnicity and OV is scarce. However, there are reports in the U.S. pointing to a relationship between racism in the health system and obstetric violence, leading to higher in rates of C-sections, obstetric morbidity and mortality in people of color and Indigenous groups [18]. Additionally, midwives in Mexico have critized current routine hospital practices, including the mistreatment of women based on social statuses, including being Indigenous [26, 27].

Lastly, women living in an urban setting had a significantly higher risk for OV, while no difference was seen for GOV. Highlands and Amazon reported higher prevalence for both subtypes of violence, compared with the Coastal region. While some of the reasons presented above could explain these residential and regional differences, further research would be needed to understand these variations.

### Methodological considerations

One of the main strengths of this study is the large study sample, representative of individual, relationship and several socio-geographical factors. The survey’s internal validity is further strengthened by the comprehensive training received by the interviewers and the thorough sampling procedures implemented to collect the data. Multiple questions covering different settings were used, contributing to a nuanced understanding of OV as violence before, during and after childbirth.

One of the main limitations of this study is that attributions to causality cannot be established, given that the data used follows a cross-sectional design. Additionally, risk for interviewer, recall and courtesy bias might be potential limitations to this study, due to the nature of the collection of data by face-to-face interviews and the exposure to violence being investigated during their lifetime. While selection bias could be a problem due to the participants´ non-response, we think that its low rate (10%) and the use of weighting in the analysis might have addressed this potential drawback.

Although several social-ecological factors were included in our analysis, other relevant factors for OV could not be considered due to their non-availability in the survey. Finally, comparison between countries might be difficult due to different methodologies and the broad definition of OV and GOV used in this study.

## Conclusion

This study aimed to determine the prevalence of OV and GOV and assess its socio-geographical distribution in Ecuador. Our results showed that 32.79% of women reported ever having experienced OV and 41.68% GOV in their lifetime.

Overall, the risk for exposure to OV was elevated in women older than 25 years, notably those between 26 and 35 and 46–55 years old with primary to secondary education, belonging to the POC, mestizo and indigenous group, who had or had had a partner, residing in urban settings and in the Highlands or Amazon regions. For GOV, the risk for exposure was also increased in women older than 25 years, particularly > 65 years old, with none to secondary education, belonging to a POC, indigenous or mestizo group, who had or had had a partner and were living in the Highlands or Amazon regions.

OV and GOV are a concerning public health issues in Ecuador. Creating awareness and a greater understanding of which groups are affected by this violence in Ecuador are an integral part of improving sexual and reproductive health and rights in Ecuador. Although the country has taken steps in the right direction, current policies and laws to protect women need to be expanded and effectively implemented to reduce OV and GOV. To achieve this, further economic resources should definitely be allocated. An intersectional approach in health research methodology, including ethnicity, would be essential to capture the complexity of women’s health experiences of structural healthcare violence. Finally, policies and training against providers’ biases in clinical care in Ecuador should be urgently implemented.

## Data Availability

The data that support the findings of this study are publicly available at: https://www.ecuadorencifras.gob.ec/violencia-de-genero/.

## Declarations

methods were carried out in accordance with the Helsinki declaration.

## References

[1] Inter-American Commission of Women. Second Hemispheric Report on the Implementation of the Belém do Pará Convention. OAS Official Records Series 2021. https://www.oas.org/en/media_center/press_release.asp?sCodigo=E-249/12. Accessed 15 May 2022.

[2] World Health Organization. The prevention and elimination of disrespect and abuse during facility-based childbirth. Geneva: WHO; 2014. https://www.who.int/publications/i/item/WHO-RHR-14.23. Accessed 16 May 2022.

[3] Jardim DMB, Modena CM (2018). Obstetric violence in the daily routine of care and its characteristics. Rev Lat Am Enfermagem.

[4] Bohren MA, Vogel JP, Hunter EC, Lutsiv O, Makh SK, Souza JP, Aguiar C, Saraiva Coneglian F, … Gülmezoglu AM. The mistreatment of women during childbirth in health facilities globally: A mixed-methods systematic review. PLoS Med. 2015;12(6): e1001847. 10.1371/journal.pmed.1001847 PMC4488322 26126110

[5] Kruk ME, Kujawski S, Mbaruku G, Ramsey K, Moyo W, Freedman LP (2018). Disrespectful and abusive treatment during facility delivery in Tanzania: a facility and community survey. Health Policy Plan.

[6] Santiago RV, Monreal LA, Rojas Carmona A, Domínguez MS. If we’re here, it’s only because we have no money… discrimination and violence in Mexican maternity wards.“ BMC Pregnancy Childbirth. 2018;18(1):244. 10.1186/s12884-018-1897-8 PMC6006746 29914421

[7] Bohren MA, Mehrtash H, Fawole B, Maung TM, Balde MD, Maya E, Thwin SS, Aderoba AK,… Tunçalp Ö. How women are treated during facility-based childbirth in four countries:a cross-sectional study with labour observations and community-based surveys. Lancet.2019;394(10210):1750-63. 10.1016/S0140-6736(19)31992-0 PMC6853169 31604660

[8] Sheferaw ED, Kim YM, van den Akker T, Stekelenburg J (2019). Mistreatment of women in public health facilities of Ethiopia. Reprod Health.

[9] Bhattacharya S, Sundari Ravindran TK (2018). Silent voices: institutional disrespect and abuse during delivery among women of Varanasi district, northern India. BMC Pregnancy Childbirth.

[10] Vedam S, Stoll K, Taiwo TK, Rubashkin N, Cheyney M, Strauss N, McLemore M, Cadena M, … GVtM-US Steering Council. The Giving Voice to Mothers study: inequity and mistreatment during pregnancy and childbirth in the United States. Reprod Health. 2019;16(1):77. 10.1186/s12978-019-0729-2 PMC6558766 31182118

[11] Homer CSE, Bohren MA, Wilson A, Vogel JP. Achieving inclusive and respectful maternity care. In Flenady V, editors. Elements of professional care and support before, during and after pregnancy. Global library of women’s medicine 2021. https://www.glowm.com/article/heading/vol-3--elements-of-professional-care-and-support%20before-during-and-after-pregnancy--achieving-inclusive-and-respectful-maternity-care/id/411763#.YkAldjXRY2x. Accessed 18 May 2022.

[12] Ravaldi C, Skoko E, Battisti A, Cericco M, Vannacci A (2018). Abuse and disrespect in childbirth assistance in Italy: A community-based survey. Eur J Obstet Gynecol Reprod Biol.

[13] Mena-Tudela D, Iglesias-Casás S, González-Chordá VM, Cervera-Gasch Á, Andreu-Pejó L, Valero-Chilleron MJ (2020). Obstetric violence in Spain (Part I): Women’s perception and interterritorial differences. Int J Environ Res Public Health.

[14] Lukasse M, Schroll AM, Karro H, Schei B, Steingrimsdottir T, Van Parys AS, Ryding EL, Tabor A, Bidens Study Group (2015). Prevalence of experienced abuse in healthcare and associated obstetric characteristics in six European countries. Acta Obstet Gynecol Scand.

[15] Lansky S, Souza KV, Peixoto ERM, Oliveira BJ, Diniz CSG, Vieira NF, Cunha RO, Friche AAL (2019). Obstetric violence: influences of the Senses of Birth exhibition in pregnant women childbirth experience. Ciên Saúde Colet.

[16] Castro R, Frías SM (2020). Obstetric violence in Mexico: results from a 2016 national household survey. Violence Against Women.

[17] Shrivastava S, Sivakami M (2020). Evidence of obstetric violence in India: an integrative review. J Biosoc Sci.

[18] Liese KL, Davis-Floyd R, Stewart K, Cheyney M (2021). Obstetric iatrogenesis in the United States: the spectrum of unintentional harm, disrespect, violence, and abuse. Anthropol Med.

[19] de Justicia M. Derechos Humanos y Cultos (2014). Codigo Orgánico Integral Penal. http://www.funcionjudicial.gob.ec/www/pdf/Codigo Organico Integral Penal.pdf. Accessed 14 May 2022.

[20] Ministry of Public Health of Ecuador. Ley de derechos y amparo del paciente [Law of Rights and Protection of the Patient]. Congreso Nacional, Gobierno de la Republica del Ecuador, Quito, Ecuador; 2014.

[21] Instituto Ecuatoriano de Estadística y Censos (INEC). Encuesta nacional sobre relaciones familiares y violencia de género contra las mujeres (ENVIGMU). https://www.ecuadorencifras.gob.ec/violencia-de-genero/. Accessed 10 May 2022.

[22] Instituto Nacional de Estadística y Censos (INEC). - Secretaría Nacional de Planificación y Desarrollo (SENPLADES). Encuesta nacional de relaciones familiares y violencia de género contra las mujeres. Ecuador, INEC, SENPLADES. https://anda.inec.gob.ec/anda/index.php/catalog/94. Accessed 10 May 2022.

[23] Instituto Nacional de Estadística y Censos (INEC). (2019b). Encuesta nacional sobre relaciones familiares y violencia de género contra las mujeres: Metodología [National survey on family relations and gender-based violence against women: methodology]. Quito: INEC; 2019. https://www.ecuadorencifras.gob.ec/documentos/web-inec/Estadisticas_Sociales/Violencia_de_genero_2019/Documento%20metodologico%20ENVIGMU.pdf. Accessed 15 May 2022.

[24] Krug EG, Mercy JA, Dahlberg LL, Zwi AB (2002). The world report on violence and health. Lancet.

[25] Consejo C, Viesca-Treviño C (2008). Ética y relaciones de poder en la formación de médicos residentes e internos: Algunas reflexiones a la luz de Foucault y Bourdieu. Bol Mex His Fil Med.

[26] Bauer GR (2014). Incorporating intersectionality theory into population health research methodology: challenges and the potential to advance health equity. Soc Sci Med.

[27] Zacher Dixon L (2015). Obstetrics in a time of violence: Mexican midwives critique routine hospital practices. Med Anthropol Q.

